# Cigarette and e-cigarette dual use and risk of cardiopulmonary symptoms in the Health eHeart Study

**DOI:** 10.1371/journal.pone.0198681

**Published:** 2018-07-25

**Authors:** Julie B. Wang, Jeffrey E. Olgin, Gregory Nah, Eric Vittinghoff, Janine K. Cataldo, Mark J. Pletcher, Gregory M. Marcus

**Affiliations:** 1 24K Data, Washington, DC, United States of America; 2 Division of Cardiology, School of Medicine, University of California, San Francisco, United States of America; 3 Physiological Nursing, School of Nursing, University of California, San Francisco, United States of America; 4 Department of Epidemiology and Biostatistics, University of California, San Francisco, United States of America; Rajiv Gandhi Centre for Biotechnology, INDIA

## Abstract

E-cigarettes are promoted as healthier alternatives to conventional cigarettes. Many cigarette smokers use both products. It is unknown whether the additional use of e-cigarettes among cigarette smokers (dual users) is associated with reduced exposure to tobacco-related health risks. Cross-sectional analysis was performed using baseline data from the Health eHeart Study, among English-speaking adults, mostly from the United States. Cigarette use (# cigarettes/day) and/or e-cigarette use (# days, # cartridges, and # puffs) were compared between cigarette only users vs. dual users. Additionally, we examined cardiopulmonary symptoms/ conditions across product use: no product (neither), e-cigarettes only, cigarettes only, and dual use. Among 39,747 participants, 573 (1.4%) reported e-cigarette only use, 1,693 (4.3%) reported cigarette only use, and 514 (1.3%) dual use. Dual users, compared to cigarette only users, reported a greater median (IQR) number of cigarettes per day, 10.0 (4.0–20.0) vs. 9.0 (3.0–15.0) (*p* < .0001), a lower (worse) median (IQR) SF-12 general health score, 3.3 (2.8–3.8) vs. 3.5 (2.8–3.9) (*p* = .0014), and a higher (worse) median (IQR) breathing difficulty score in the past month, 2.0 (1.0–2.0) vs. 1.0 (1.0–2.0) (*p* = .001). Of the 19 cardiopulmonary symptoms/ conditions, having a history of arrhythmia was significantly different between cigarette only users (14.2%) and dual users (17.8%) (*p* = .02). In this sample, dual use was not associated with reduced exposure to either (i) cigarettes, compared to cigarette only users or (ii) e-cigarettes, compared to e-cigarette only users. E-cigarette only use, compared to no product use, was associated with lower general health scores, higher breathing difficulty scores (typically and past month), and greater proportions of those who responded ‘yes’ to having chest pain, palpitations, coronary heart disease, arrhythmia, COPD, and asthma. These data suggest the added use of e-cigarettes alone may have contributed to cardiopulmonary health risks particularly respiratory health risks.

## Introduction

The harmful health effects of tobacco use are well-established, including an array of cardiovascular and pulmonary diseases and cancers [1]. Nearly 15% of the U.S. adult population smoke cigarettes [2]. Although most smokers want to quit, and repeatedly make quit attempts, they frequently relapse even with the assistance of evidence-based cessation therapeutics [3]. Since the emergence of e-cigarettes (and vapes), cigarette smokers have initiated e-cigarette use with the intention to reduce or quit smoking conventional cigarettes [4–11]. E-cigarette companies have promoted e-cigarettes as less harmful than cigarettes, and as a cessation aid, although without formal approval by the US Food and Drug Administration to make such therapeutic claims [12]. As of 2014, nearly 4% of adults reported e-cigarette use every day or on some days [13] and 59% of e-cigarette user also reported cigarette use [14]. It is unclear whether dual users are smoking fewer cigarettes, given the additional source of nicotine via e-cigarettes, compared to those who only smoke cigarettes. The present study examines exposure to tobacco- and e-cigarette-related toxicants and health risks by product use, particularly, whether the additional use of e-cigarettes among cigarette smokers, or dual use, was associated with benefits such as reduced risk exposure.

A major barrier to quitting smoking is the addictiveness of nicotine, which is compounded by cigarette engineering that maximizes the addictive potential of delivered nicotine [15–17]. A longitudinal study among the US population reported smokers who made a quit attempt were more likely to use e-cigarettes over current FDA-approved pharmacotherapies [18]. Smokers’ preferences for e-cigarettes over approved cessation methods might be explained by the novelty of e-cigarettes, although it has also been shown that products such as nicotine patches, gum, and inhalers were less efficient than e-cigarettes in delivering nicotine [19]. Epidemiologic data indicate that approximately 22% of e-cigarette users were recent former cigarette smokers (< 1 year) and 16% were current cigarette smokers [13]. These data suggest that some smokers may have quit with the aid of e-cigarettes. A substantial subset of the population however are dual users, and, unless they quit smoking cigarettes, are exposed to harmful substances associated with cigarettes and potentially e-cigarettes.

Hypothetically, among dual users, the addition of nicotine from e-cigarettes should supplant nicotine levels otherwise met by smoking cigarettes and thereby reduce their cigarette intake. However, e-cigarettes deliver lower levels of plasma nicotine compared to conventional cigarettes [19–21], which could leave dual users unsatisfied and likely to titrate their nicotine intake to reach satiety. Therefore, it is unclear whether dual users are smoking fewer cigarettes per day and reducing their exposure to tobacco smoke and nicotine, or if they are smoking the same amount and merely introducing more toxicants and nicotine from the added use of e-cigarettes. Experimental studies have found a link between e-cigarette use and effects on pulmonary function, including irritation and restriction of airways, from the effects of propylene glycol, which is a main ingredient of e-liquids [22]. The synergistic effect of dual use is largely unknown with respect to nicotine dependence and both short-term and long-term health effects. Epidemiologic data on dual use and health is scarce and warrants further investigation.

The Health eHeart Study is an ongoing internet-based study aims to collect and analyze mobile and digital health data, with an enrollment goal of one million participants worldwide, to discover novel approaches to preventing and treating heart disease [23]. It includes assessment of cigarette and e-cigarette use *and* health symptoms and conditions. The primary purpose of the present study was to assess whether dual users smoke fewer/more cigarettes per day, and therefore might have lower/higher risk exposure from conventional cigarettes, compared to those who smoke only cigarettes. A secondary aim was to compare differences in the prevalence of pulmonary and cardiovascular health symptoms and conditions across product use groups particularly between cigarette only users and dual users. These findings can inform whether dual users might be at greater exposure to toxicants and health risks, and therefore, might require more tailored guidance for quitting cigarettes and e-cigarettes.

## Methods

### Study design

We conducted cross-sectional analyses using data from the Health eHeart Study, which includes English-speaking adults aged 18 years and over. Health eHeart is an on-going longitudinal cardiovascular cohort study. For the present analysis, we analyzed available baseline data from March 8, 2013 (beginning of recruitment) to March 1, 2017. Participants are recruited worldwide via the lay press, promotional events, word-of-mouth, social media, e-mail, and clinic visits and invited to complete a set of web-based surveys at baseline and every 6 months for 2 years. Majority of participants were from the United States. Survey topics include basic and social demographics, family history, medical history, activity level and other lifestyle habits, including cigarette and e-cigarette use, and technology use. In the present study, we examined participants’ product use: (i) no product use (neither), (ii) cigarettes only, (iii) e-cigarettes only, or (iv) both (dual use), including e-cigarette and cigarette dose. Additionally, we examined relationships between product use and health outcomes including cardiopulmonary symptoms and conditions. The Health eHeart Study was approved by the University of California, San Francisco Committee on Human Research. All participants provided informed consent through the internet.

### Measures

#### Demographics, lifestyle, and well-being

Basic and social demographics included age, sex, race/ethnicity, education, income, familial relationships, and technology use. Lifestyle and well-being were measured using validated survey instruments, which included the 7-item International Physical Activity Questionnaire [24], 9-item Patient Health Questionnaire (mood) [25], and 7-item General Anxiety Disorder Scale [26].

#### Product categories: Neither, e-cigarettes, cigarettes, and dual use

Participants who responded (1) “yes” to “Have you ever smoked cigarettes regularly (at least 1 cigarette per day and a total of 100 cigarettes in your lifetime)?” and (2) “every day” or “some days” to “Do you smoke now?” were defined as (current) *cigarette users*. Number of cigarettes was assessed with the question “On average, how many cigarettes per day do you smoke?” Those who responded, “every day” or “some days” to “Do you now use e-cigarettes” were defined as (current) *e-cigarette users*. E-cigarette dose was asked in several ways: (1) “On how many of the past 30 days did you use an e-cigarette?”; (2) “On average, [on those days you used e-cigarettes], about how many cartridges or refills did you usually use each day?” Those who qualified as both a cigarette user and e-cigarette user were defined as *dual users*. No product use was categorized as *neither*.

#### Medical symptoms & conditions

Medical symptoms and conditions that might be associated with either cigarette or e-cigarette dual use were included for analysis. The SF-12 General Health Questionnaire was used to calculate an overall health score [27]. Two separate items assessed breathing difficulty: (1) “This is a scale that asks you to rate the difficulty of your breathing. It starts at number 0 (nothing at all) where your breathing is causing you no difficulty at all and progresses through to number 10 (maximal) where your breathing difficulty is maximal. How much difficulty does your breathing cause you *typically*?” and (2) “In thinking about your breathing, and any difficulties you may have with your breathing, what level of difficulty best describes your breathing normally over the *past month*?” Response options were: (i) I only get breathless with strenuous exercise; (ii) I get short of breath when hurrying on level ground or walking up a slight hill; (iii) On level ground, I walk slower than people of the same age because of the breathlessness or have to stop for breath when walking at my own pace; (iv) I stop for breath after walking about 100 yards or after a few minutes on level ground; and (v) I am too breathless to leave the house or I am breathless when dressing.

Other medical symptoms and conditions included chest pain, “Have you had any pain, discomfort or pressure in your chest anytime over the past year.” “Have you ever been told by a doctor or nurse that you have, or have been treated for, any of the following conditions (in the past or currently)?”: palpitations, loss of consciousness or syncope, high blood pressure or hypertension, high cholesterol, diabetes, coronary artery disease/ angina, heart attack, blocked arteries (legs), blood clots (veins or lungs), congestive heart failure, stroke or TIA (transient ischemic attack), enlarged heart, atrial fibrillation, arrhythmia, sleep apnea, COPD, asthma, or cardiac arrest.

#### Statistical analysis

Descriptive analysis was performed for all variables. Means (SD) were calculated for parametric continuous variables and compared using *t* tests, linear regression, ANOVA, or ANCOVA; and percentages for categorical variables using Chi-square tests. The distributions of continuous variables that were not normally described are summarized as medians with interquartile ranges (IQR), and those described were compared using the Wilcoxon-Mann-Whitney test. Basic demographic variables and lifestyle and well-being factors were compared across e-cigarette, cigarette, and dual users. In separate models, we compared (i) number of cigarettes smoked per day between cigarette only users and dual users and (ii) e-cigarette dose variables between e-cigarette only users and dual users (i.e., number of days in the past 30 days, number of cartridges/refills per day, and number of puffs per day). Multivariate models adjusted for age, sex, race, education, physical activity, PQH score, and GAD score.

In the health symptoms/conditions analysis, we compared general health scores and two separate breathing difficulty scores (*typically* and *in the past month*), and frequency of “yes” responses to ever or currently having a symptom or condition, between cigarette only users and dual users. Additional analysis compared the same set of health symptoms/ conditions across mutually exclusive product groups: neither (reference), e-cigarettes only, cigarettes only, and both. Separate ANCOVA were fitted for general health, difficulty breathing (typically), difficulty breathing (past month), chest pain, palpitations, lost consciousness or syncope, high blood pressure, high cholesterol, diabetes, coronary artery disease, heart attack, blocked arteries in the legs, blood clots, congestive heart failure, stroke, enlarged heart, atrial fibrillation, arrhythmia, sleep apnea, COPD, asthma, and cardiac arrest. Multivariate models were adjusted for age, sex, race, education, cigarettes per day, coronary artery disease, congestive heart failure, and COPD. All statistical analyses were performed using SAS Version 9.4 (SAS Institute, Cary NC).

## Results

### Sample characteristics

In the ongoing Health eHeart Study, the 39,747 participants who had complete baseline data at the time of analysis were included in our study. In this sample, participants were mostly non-Hispanic White, female, and with particularly high levels of education. A total of 573 (1.4%) reported using e-cigarettes only, 1,693 (4.3%) cigarettes only, and 514 (1.3%) reported dual use (Table 1). Among e-cigarette only users, 118 (21%) reported that they had never smoked even one cigarette in their lifetime. E-cigarette only users were younger, more likely to be male, Asian, and have a high school level of education. Cigarette only users were more likely to be male, in the “other” race/ethnicity, and have less than a high school level of education. Dual users were most likely to be Asian and have less than a high school level of education. Dual users reported the lowest physical activity levels, followed by cigarette only users, then e-cigarette only users. Dual users also exhibited higher depression and higher anxiety scores compared to cigarette only users.

**Table 1:** Prevalence of e-cigarette only, cigarette only, and dual use in the past 30 days by demographic characteristics and lifestyle and well-being factors in the health eheart study, N = 39,747*.

		E-cigarette use only	Cigarette use only	Dual use	
		(N = 573)	(N = 1,693)	(N = 514)	
	N	% or median (IQR)	% or median (IQR)	% or median (IQR)	*p*-value
**Overall**	39,747	1.4	4.3	1.3	
**Demographics**					
Age, years	39,645	41.4 (18)	45 (21)	46 (18)	< .0001*
Sex					
Female	27,600	1.1	3.9	1.3	< .0001*
Male	12,047	2.3	5.0	1.4	
Race/ ethnicity					
Non-Hispanic White	32,302	1.4	3.9	1.3	.99
Hispanic	2,761	2.3	5.4	1.1	
Black/African-American	2,014	0.7	5.3	1.3	
Asian	1,723	3.8	4.7	3.5	
Other	445	2.7	12.8	2.3	
Education					
< High school	171	1.8	21.1	4.1	.0007*
High school graduate	2,166	2.9	7.9	3.1	
Some college	8,169	2.1	6.2	2.0	
College graduate	11,466	1.4	2.9	0.8	
≥ Post graduate	12,419	0.6	2.1	0.4	
**Lifestyle and Well-being**					
Physical activity (MET-min/week)	20,094	2,538.5 (2,973)	2,493.0 (3,661)	2,377.5 (3,222)	.0007*
Mood (PHQ-9 score, past 2 weeks)	39,543	6.0 (8.0)	7.0 (8.0)	8.0 (9.0)	< .0001*
Anxiety (GAD-7 score, past 2 weeks)	39,539	5.0 (8.0)	6.0 (9.0)	7.0 (9.0)	.0008*

* Available responses were included in the analysis; physical activity was collected in a subset of the Health eHeart Study population

### E-cigarette and cigarette use

**Fig 1:**
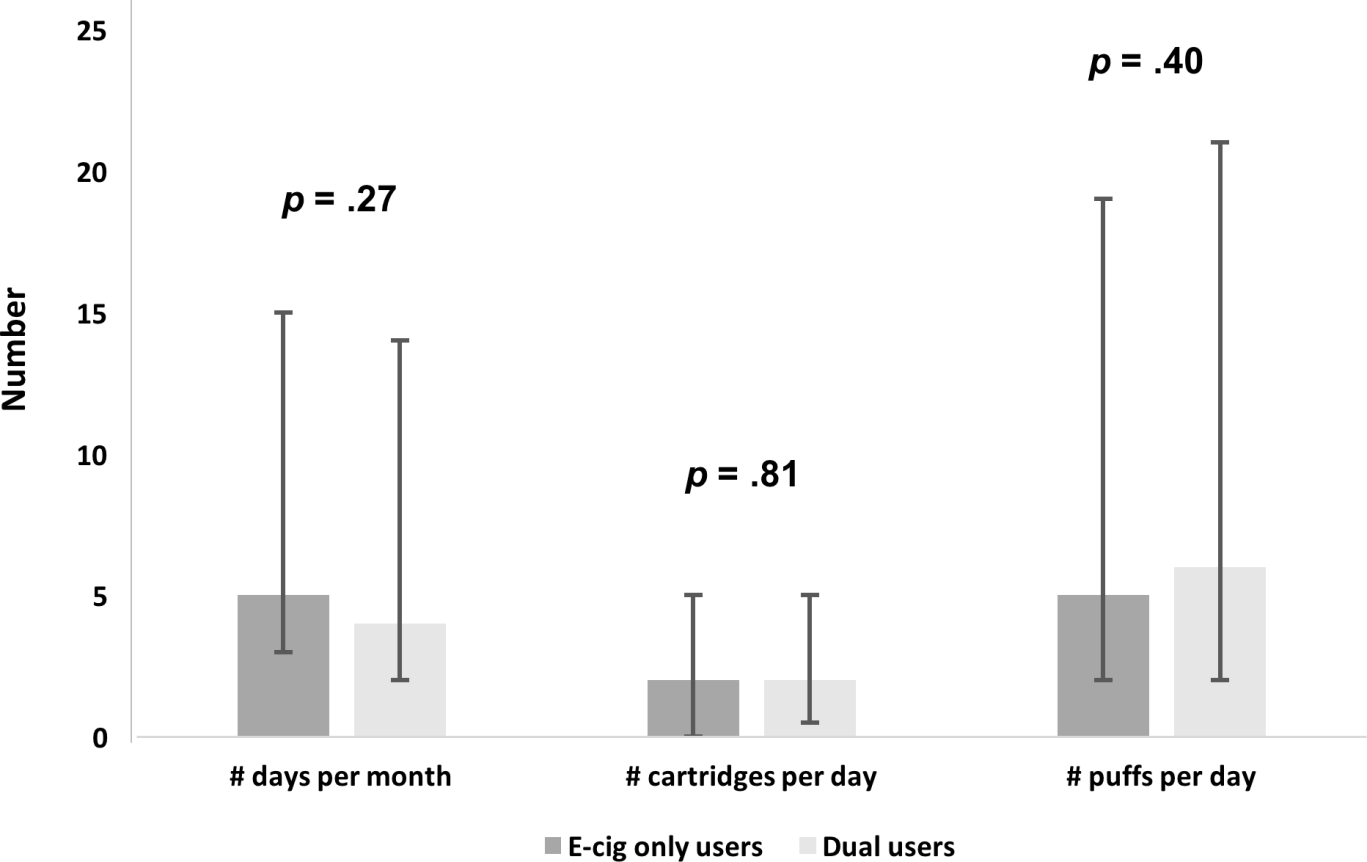
E-cigarette use dose measures among e-cigarette only users and dual users. (a) # days per month was defined as “number of days of e-cigarette use in the past 30 days” (b) # cartridges per day: “number of e-liquid cartridges/refills used per day” (c) # puffs per day: “number of puffs off an e-cigarette per day.” Error bars denote the interquartile range.

**Fig 2:**
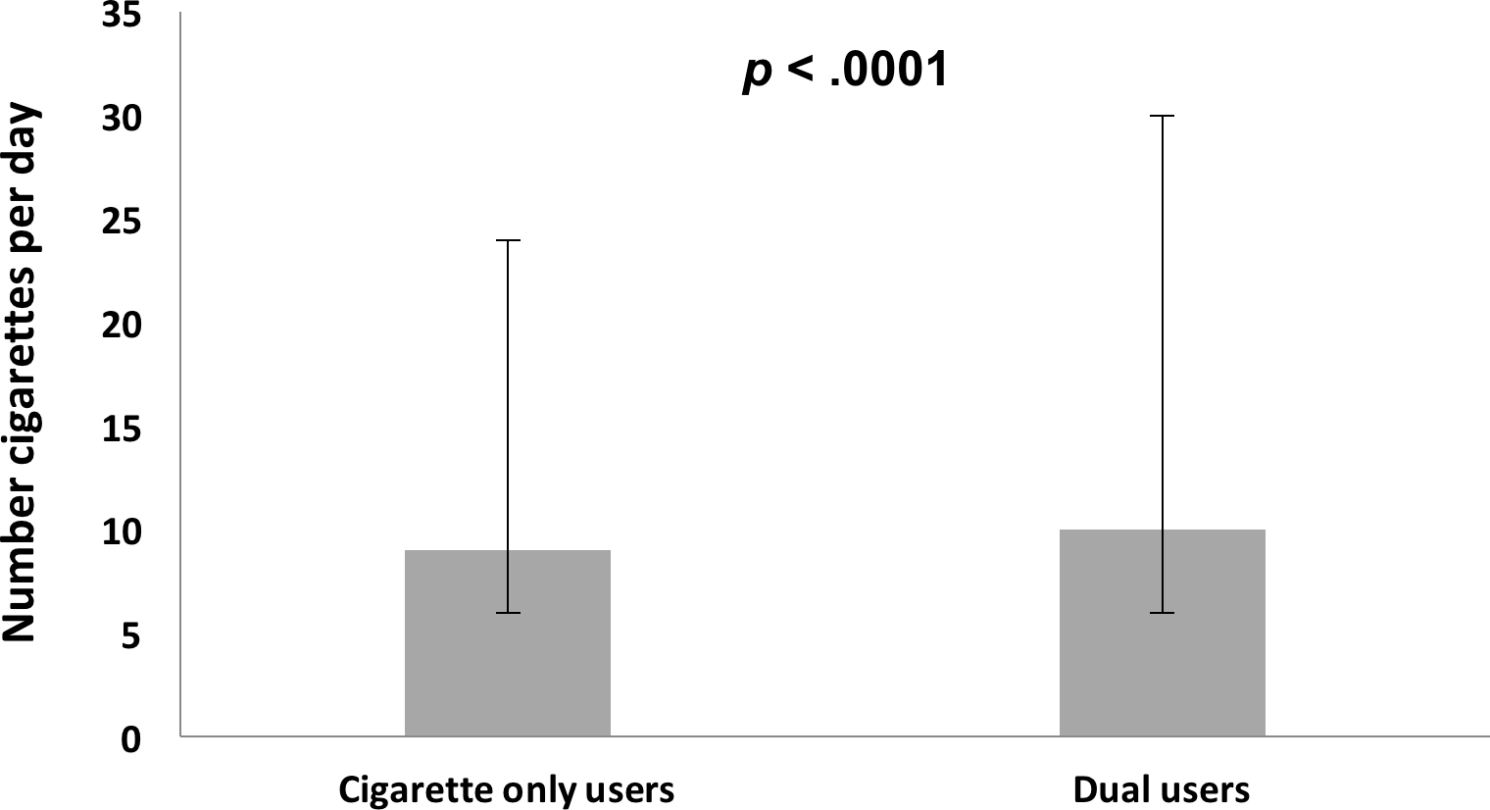
Median number of cigarettes smoked per day among cigarette only users and dual users. Dual use was associated with a slightly higher median number of cigarettes smoked per day (*p* < .0001). Error bars denote the interquartile range.

Among e-cigarette users, 53% reported using e-cigarettes only, and 47% were dual users. There were no significant differences in e-cigarette dose measures in those who reported e-cigarette only use and dual use for number of: (i) days of e-cigarette use in the past month, (ii) cartridges/ refills per day, or (iii) puffs per day (Fig 1). For those who reported cigarette use, 77% reported using cigarettes only and 23% were dual users. Dual use was associated with a slightly higher median number of cigarettes smoked per day (10.0 cigarettes per day, IQR = 3.0–15.0) compared to those who reported smoking only cigarettes (9.0 cigarettes per day, IQR = 4.0–20.0), and this difference was statistically significant after adjusting for covariates (*p* < .0001) (Fig 2).

### Medical symptoms and conditions

**Fig 3:**
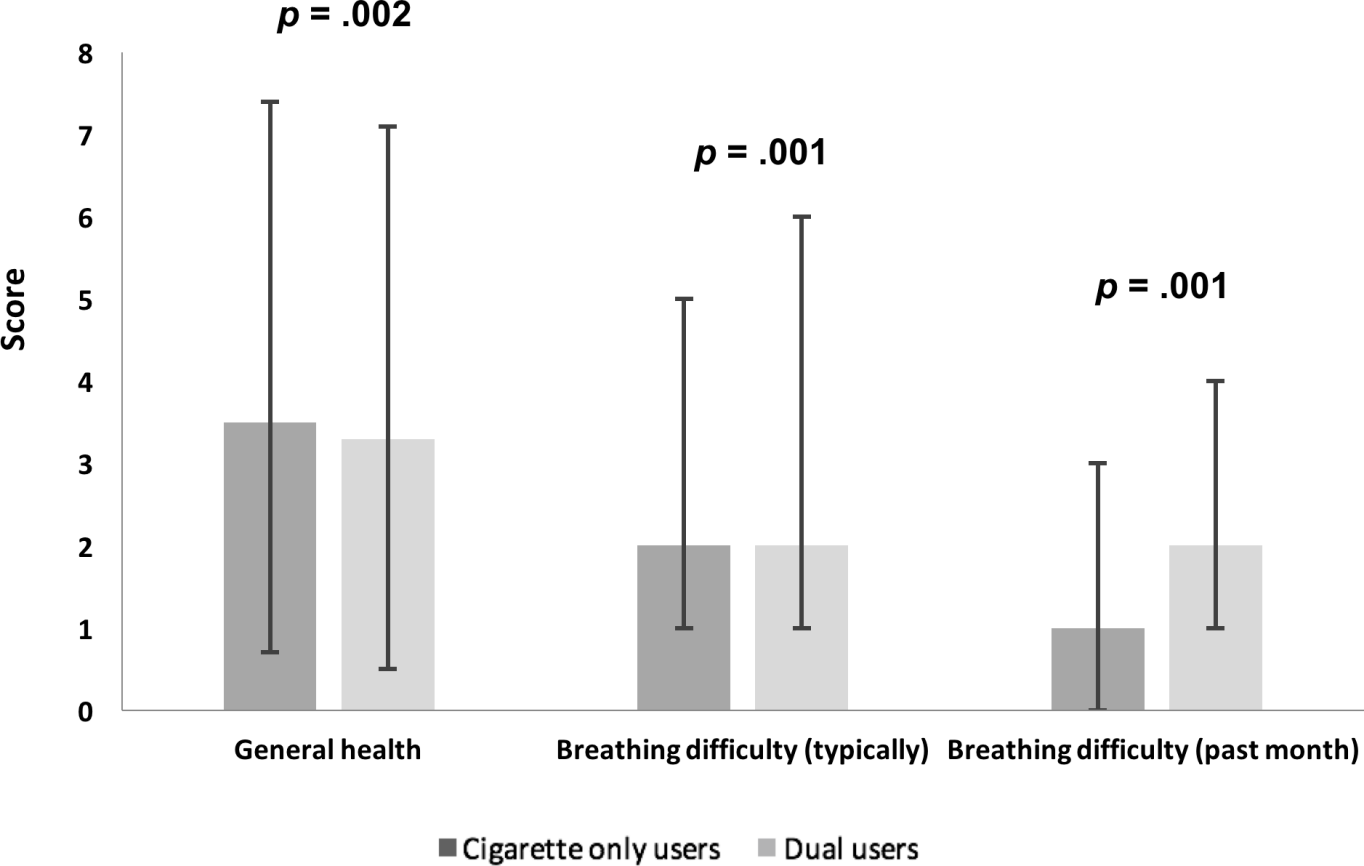
Median SF-36 general health scores, breathing difficulty “typically” scores, and breathing difficulty in the “past month” scores, among cigarette only users and dual users. Dual use was associated with lower (poorer) general health scores (ADJ *p* = .002) and higher (poorer) past month breathing difficulty scores (ADJ *p* = .001). Models adjusted for age, sex, race, education, cigarettes per day, coronary artery disease, congestive heart failure, and COPD. Error bars denote the interquartile range.

**Fig 4:**
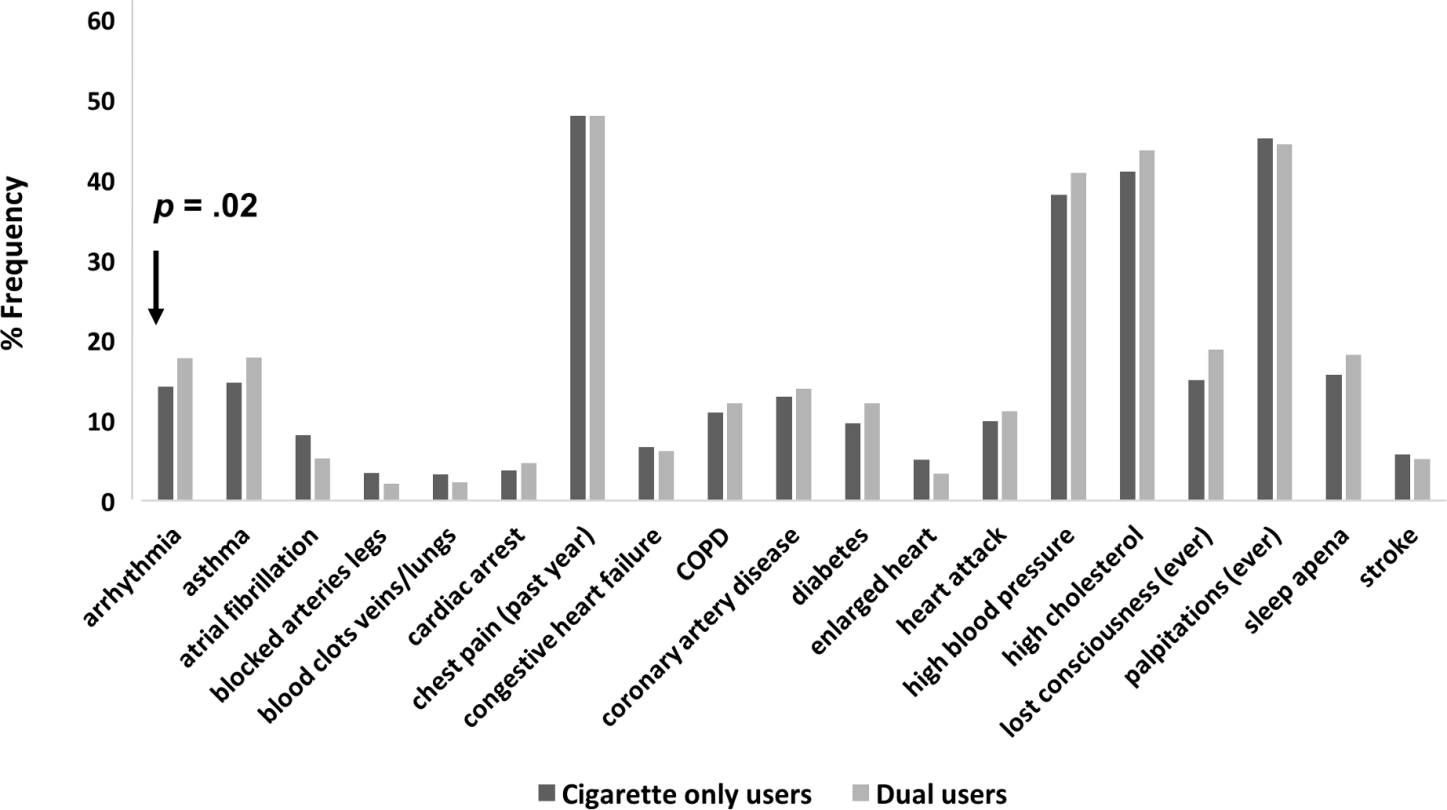
Percent of “yes” responses to past or current medical symptoms or conditions among cigarette only users and dual users. The only statistically significant difference between cigarette only users and dual users was arrhythmia (ADJ *p* = .02). Models adjusted for age, sex, race, education, cigarettes per day, and as needed, coronary artery disease, congestive heart failure, and COPD.

Compared to those who only smoked cigarettes, dual users exhibited worse median general health scores and breathing scores. Dual use, as compared to smoking cigarettes only, was associated with lower general health scores (worse) (UADJ: ß = -.04, 95% CI: -.06 to -.01, *p* = .003; ADJ: ß = -.02, 95% CI: -.05 to .01, *p* = .002), comparable breathing difficulty scores *typically* (UADJ: ß = .003, 95% CI: .-007 to .01, *p* = .53; ADJ: ß = -.0009, 95% CI: -.01 to .01, *p* = .001), and higher (worse) breathing difficulty scores in the *past month* (UADJ: ß = .02, 95% CI: -.009 to .04, *p* = .21; ADJ: ß = .01, 95% CI: -.02 to .04, *p* = .001), after adjusting for age, sex, race, education, cigarettes smoked per day, coronary artery disease, congestive heart failure, and COPD (Fig 3). In examining the 19 cardiopulmonary conditions and risk factors between the two groups, only having a history of an arrhythmia was found to be significantly different between cigarette only users (14.2%) and dual users (17.8%) after adjusting for covariates (*p* = .02) (Fig 4).

E-cigarette use alone was associated with risk of health symptoms and conditions. Compared to those who reported no product use (reference group), those who reported e-cigarette only use, had lower (worse) general health scores, higher (worse) breathing difficulty scores, both *typically* and in the *past month*, and were more likely to report chest pain, palpitations, coronary artery disease, arrhythmia, COPD, and asthma (Table 2).

**Table 2:** Medical symptoms and conditions by product use, N = 34,279.

	Neither	E-cigarette Use Only	Cigarette Use Only	Dual Use	*p*-value unadjusted	*p*-value adjusted*
	N (mean or %)	N (mean or %)	N (mean or %)	N (mean or %)		
General health score	36,506 (3.8)	588 (3.5)	1626 (3.4)	480 (3.3)	< .0001	< .0001
Breathing difficulty, typically	31,924 (1.8)	483 (2.3)	1274 (2.5)	365 (2.5)	< .0001	< .0001
Breathing difficulty, past month	31,909 (1.3)	483 (1.4)	1273 (1.6)	365 (1.6)	< .0001	< .0001
Chest pain	10,730 (32.9%)	232 (47.3%)	685 (52.0%)	197 (51.7%)	< .0001	< .0001
Palpitations	14,477 (44.5%)	266 (55.3%)	721 (54.8%)	211 (55.5%)	< .0001	.002
Lost consciousness/ syncope	4,582 (14.1%)	413 (15.7%)	1119 (15.0%)	309 (18.7%)	.01	.06
High blood pressure	11,931 (36.5%)	167 (34.0%)	507 (38.2%)	157 (40.9%)	.07	.02
High cholesterol	13,812 (42.3%)	158 (32.1%)	544 (41.1%)	167 (43.7%)	.21	.29
Diabetes	2,578 (7.9%)	33 (6.7%)	129 (9.7%)	47 (12.2%)	.0005	.02
Coronary artery disease	3,248 (9.9%)	54 (10.9%)	174 (13.0%)	54 (14.0%)	< .0001	< .0001
Heart attack	1,931 (5.9%)	28 (5.7%)	132 (9.9%)	43 (11.2%)	< .0001	.67
Blocked arteries in the legs	550 (1.7%)	9 (1.8%)	46 (3.5%)	8 (2.1%)	< .0001	.34
Blood clots	1,104 (3.4%)	19 (3.9%)	43 (3.3%)	9 (2.3%)	.47	.58
Congestive heart failure	1,303 (4.0%)	16 (3.2%)	89 (6.7%)	24 (6.2%)	< .0001	.09
Stroke	1,218 (3.7%)	13 (2.6%)	77 (5.8%)	20 (5.2%)	.0004	.53
Enlarged heart	1,330 (4.1%)	20 (4.1%)	68 (5.1%)	13 (3.4%)	.32	.23
Atrial fibrillation	2,732 (8.4%)	20(4.1%)	108 (8.2%)	20 (5.3%)	.03	.91
Arrhythmia	4,444 (13.8%)	74 (15.1%)	183 (14.2%)	66 (17.8%)	.08	.03
Sleep apnea	4,260 (13.3%)	75 (15.8%)	195 (15.4%)	63 (17.2%)	.002	.58
COPD	1,209 (3.7%)	33 (6.7%)	146 (11.0%)	47 (12.2%)	< .0001	.001
Asthma	3,356 (10.3%)	81 (16.5%)	195 (14.7%)	69 (17.9%)	< .0001	.003
Cardiac arrest	697 (2.1%)	7 (1.4%)	50 (3.8%)	18 (4.7%)	< .0001	.39

* Adjusted models included age, sex, race, education, cigarettes per day, coronary artery disease, congestive heart failure, and COPD

## Discussion

In the Health eHeart Study, there was no evidence of reduced exposure to cigarettes, e-cigarettes, or health risks among dual users. These data suggest that, in this sample, the added use of e-cigarettes did not appear to supplant nicotine levels that might have otherwise been delivered via combustible cigarettes. An alternative explanation is that those with higher baseline nicotine dependence, who typically smoke more cigarettes per day, were more likely to be dual users. Greater nicotine dependence among dual users might explain why some studies have shown that e-cigarettes were not associated with reduced smoking or quitting smoking [28–33]. In this study, dual use was associated with smoking a median of one more cigarette per day compared to those who smoked only cigarettes. This was a small and statistically significant difference. Dual use was also associated with poorer general health and greater breathing difficulty in the past month compared to those who smoked only cigarettes. Given the rather small differential in the median number of cigarettes smoked per day between dual users and cigarette only users, the role specifically that of e-cigarettes in dual users warrants further investigation. These data provide epidemiologic evidence that dual use and e-cigarette use alone were associated with greater cardiopulmonary health risks. More studies are needed to examine both short-term and long-term health effects of e-cigarette use and potential synergistic effects of dual use.

### Dual use and cigarette/e-cigarette exposure

In the present study, even with the use of e-cigarettes, dual users did not smoke fewer cigarettes than those who smoked only cigarettes. It may be possible that the added nicotine from e-cigarettes did not satisfy dual users’ nicotine levels enough to reduce their usual cigarette intake. Studies have shown that e-cigarettes deliver lower levels of plasma nicotine than conventional cigarettes [20, 21], although more experienced e-cigarette users can also adjust their devices and e-liquid nicotine concentrations to achieve their desired nicotine levels [19, 22]. In the Health eHeart Study sample, there were no detectable differences in e-cigarette use dose, as measured by number of days, cartridges/ refills, and puffs per day, between those who reported using e-cigarettes only and dual users. Overall, dual users had greater exposure to toxicants from using both cigarettes and e-cigarettes. This finding is consistent with a study by Shahab et al. (2017), where exposure to carcinogens and toxins was not substantially reduced in dual users compared to those who smoked only cigarettes [34].

In the Health eHeart Study sample, one fifth of e-cigarette users reported never smoking cigarettes, whereas the remaining majority of e-cigarette users were former cigarette smokers. This is consistent with a study by Zhu et al. (2017), which reported a significant association between a substantial increase in e-cigarette use among cigarette smokers and smoking cessation rates at the US population level [35]. These data suggest that some cigarette users, perhaps those with lower nicotine dependence, might have successfully quit smoking cigarettes with the aid of e-cigarettes. However, dual use was not associated with reduced risk of tobacco exposure compared to smoking cigarettes alone. Further, the added exposure to e-cigarette toxicants, including more nicotine, might have adverse health outcomes.

### Dual use, nicotine dependence, and health

Dual users are exposed to toxicants from cigarette smoke, e-cigarette vapor, and nicotine from both products. In the Health eHeart study, dual users were more likely to report lower general health scores and greater breathing difficulty. The health risks of first- and secondhand cigarette smoke exposure are well-established [1], and the evidence for e-cigarettes on pulmonary and cardiovascular health risks is growing. In the present study, compared to those who did not use either e-cigarettes or cigarettes, participants who reported e-cigarette use alone were more likely to report both short-term and long-term pulmonary symptoms and conditions, including breathing difficulty, asthma, and COPD. Compared to those who used e-cigarettes alone, participants who reported dual use had even greater risk of breathing difficulty, asthma, and COPD. These findings support other studies to suggest that e-cigarettes may have adverse health effects on the respiratory system. Previous studies have reported e-cigarette vapor contain aerosols and other particles, including propylene glycol, that are associated with increased risk of airway irritation and resistance [36–39]. Studies from animal and basic science models have consistently shown specific e-cigarette liquid flavorings contain respiratory toxins [40–43]. Compared to those who reported neither e-cigarette or cigarette use, those who reported e-cigarette only use were more likely to report chest pain, palpitations, coronary artery disease, and an arrhythmia, which supports findings from other studies where e-cigarette use was associated with cardiac conditions such as arrhythmias and hypertension [44]. These conditions may be attributed to the increased exposure to nicotine (from e-cigarettes), which itself is associated with acute cardiovascular events and accelerated atherogenesis [45, 46].

These study findings provide early epidemiologic evidence to suggest that dual users are at higher risk of breathing difficulty and arrhythmias, and that this increased risk is likely attributable to e-cigarette use or the potential combined effect of cigarette and e-cigarette use. More studies are needed to elucidate the relationship between dual use and both short- and long-term health outcomes. In the meantime, we recommend dual users treat e-cigarettes (at the very least) like other nicotine replacement pharmacotherapies and recognize the potential for increased nicotine exposure. There is also the added risk of exposure to e-cigarette toxicants and the potential synergistic effect of dual use on health. Therefore, dual users are encouraged to stop smoking cigarettes and e-cigarettes to minimize tobacco and e-cigarette-related health risks.

### Limitations

The cross-sectional nature of the present analysis limits causal inferences that can be made from these results. The timing of e-cigarette initiation was not available. The Health eHeart Study sample was not representative of the general US population, which could induce selection bias, and therefore limits the generalizability of our findings. For example, prevalence rates for cigarettes and e-cigarettes were lower in Health eHeart Study participants compared to the general US population. This discrepancy is likely due to higher proportions of Health eHeart Study participants, who were female, older age, and those with particularly high levels of education, which are factors associated with lower smoking prevalence rates in the general US population [47].

A validated measure of e-cigarette dose was not available, nor does one exist for comparison, which limits our ability to accurately assess frequency, quantity, and type of e-cigarette/ e-liquid use. The wide variation in e-cigarette/ vape devices, e-liquids, settings, and individual uptake that influence nicotine delivery and absorption add to the complexity in measuring e-cigarette dose [48]. In addition, it is important to mention that self-reported outcomes might result in recall bias, although the participants in this study did not necessarily know which predictors (such as e-cigarette use or smoking as opposed to all of the covariates collected in many surveys in the Health eHeart Study) would be used to assess relationships with which symptoms. The Health eHeart Study does not include assessment of baseline nicotine dependence levels. It is uncertain whether smokers with greater nicotine dependence, which is positively associated with the total number of cigarettes smoked per day and greater difficulty quitting smoking [49], were more likely to use e-cigarettes, which might explain why dual use was not associated with smoking fewer cigarettes or reduced health risks. For example, smokers who have had greater difficulty quitting smoking, prolonged exposure to tobacco smoke, and prevalence of respiratory issues, could have been more inclined to initiate e-cigarette use with the intention to reduce or quit smoking conventional cigarettes. More epidemiologic studies, including longitudinal studies, are needed to further examine dual use, exposure to cigarette and e-cigarette toxicants, and health outcomes, while accounting for baseline nicotine dependence levels.

## Conclusions

These data demonstrate that, among dual users, the use of e-cigarettes was not associated with less exposure to tobacco smoke or health risks. It provides epidemiologic evidence specifically linking e-cigarettes, and possibly a synergistic relationship between cigarettes and e-cigarettes, to having adverse pulmonary and cardiovascular health symptoms/conditions. Many smokers are adopting e-cigarettes for smoking cessation, which warrants effective strategies to help dual users wean off both cigarettes and e-cigarettes.
